# Supplementary figures and images for: High-resolution genetic mapping reveals cis-regulatory and copy number variation in loci associated with cytochrome P450-mediated detoxification in a generalist arthropod pest
Source: PLoS Genet. 2021 Jun 21;17(6):e1009422. doi: 10.1371/journal.pgen.1009422 (PMC8248744; doi:10.1371/journal.pgen.1009422)

— susceptible (control)  
— pyflubumide-selected

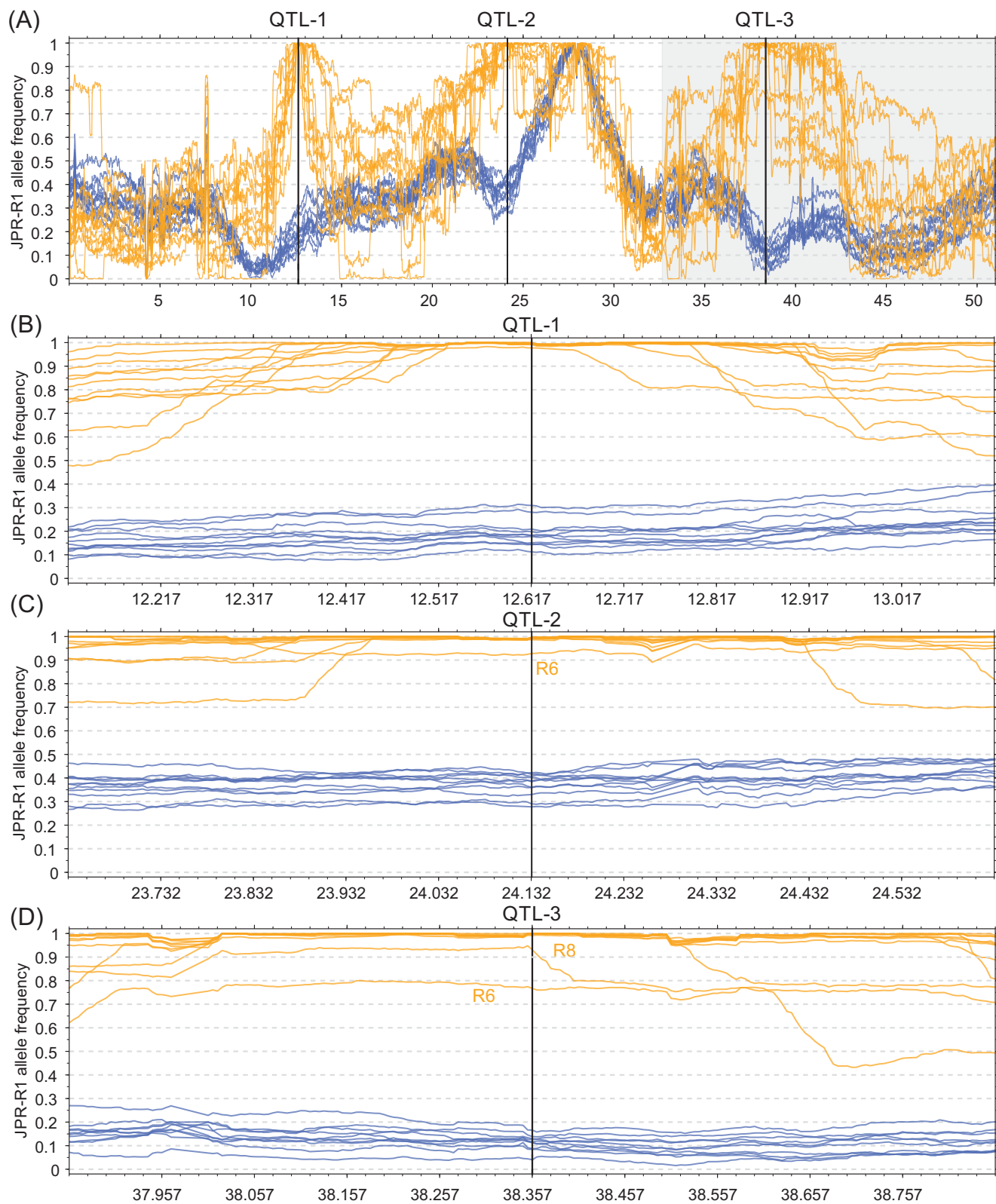

Supplement: S1 Fig — (A) Frequencies of JPR-R1 alleles in the susceptible (control) and pyflubumide-selected populations across the three QTL, as assessed in a sliding window analysis (see also Fig 4A). Chromosomes are ordered by decreasing length and are indicated by alternating shading. (B-D) Frequencies of JPR-R1 alleles in the susceptible (control) and pyflubumide-selected populations at QTL-1, QTL-2, and QTL-3, respectively. Pyflubumide-selected populations that do not show near-fixation levels of JPR-R1 allele frequencies at the averaged BSA peaks are indicated by their identifier. For all panels, coverage is color-coded according to treatment (legend, top). Vertical lines indicate the locations of the averaged BSA peaks. (PDF) [file pgen.1009422.s001.pdf]

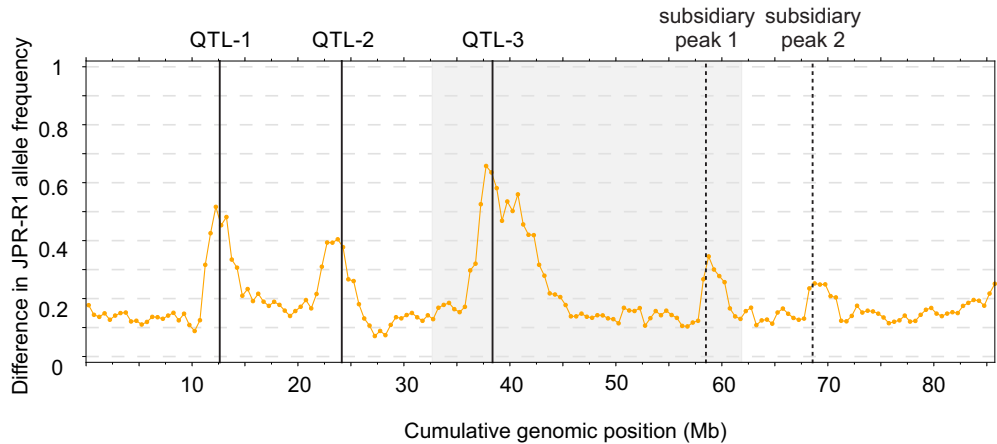

Supplement: S2 Fig — Chromosomes are ordered by decreasing length and are indicated by alternating shading. Vertical lines indicate the locations of the three QTL, whereas vertical dashed lines indicate the two subsidiary peaks (Fig 4). (PDF) [file pgen.1009422.s002.pdf]

(A) *CYP392A16*

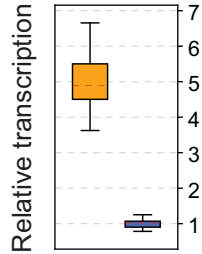

(B) *CPR*

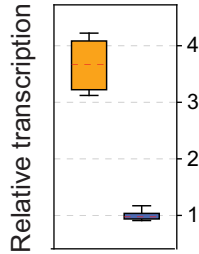

(C) *CPR*

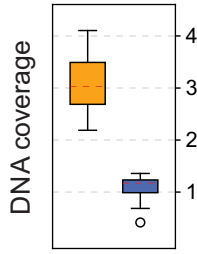

pyflubumide-selected populations  
susceptible (control) populations

Supplement: S3 Fig — CYP392A16 (A) and CPR (B) expression in pyflubumide-selected populations relative to the mean RNA abundance in the susceptible control populations. (C) DNA coverage of CPR in pyflubumide-selected and control populations, relative to single-copy VGSC. DNA coverage was estimated by quantitative PCR analysis of the segregating populations, confirming estimates using short-read coverage (Fig 5). Panels are color-coded according to treatment. (PDF) [file pgen.1009422.s003.pdf]

JPR-R1      WasX  
pyflubumide-selected      susceptible (control)

(A)

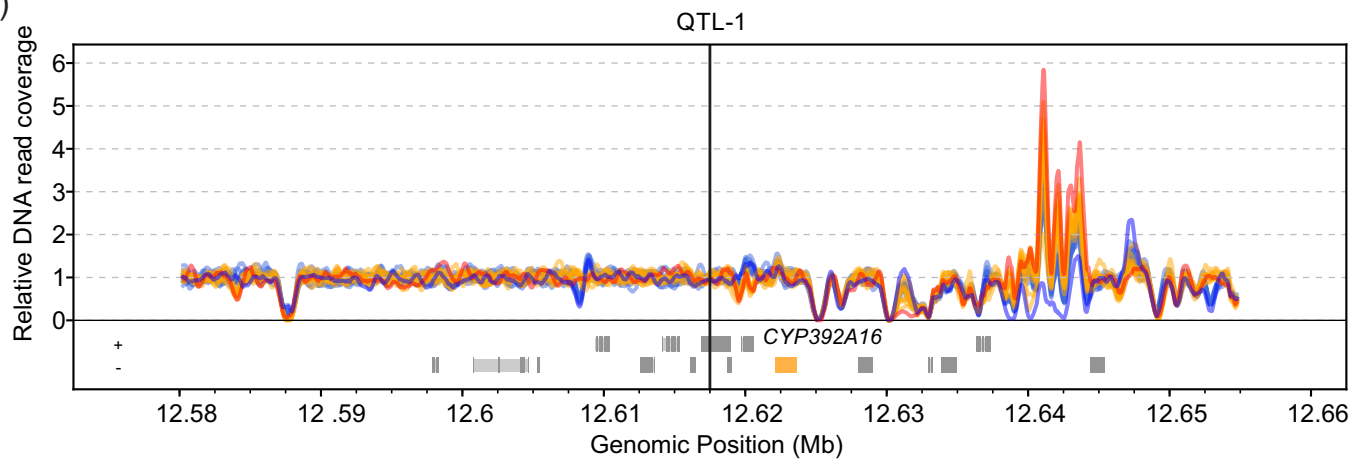

(B)

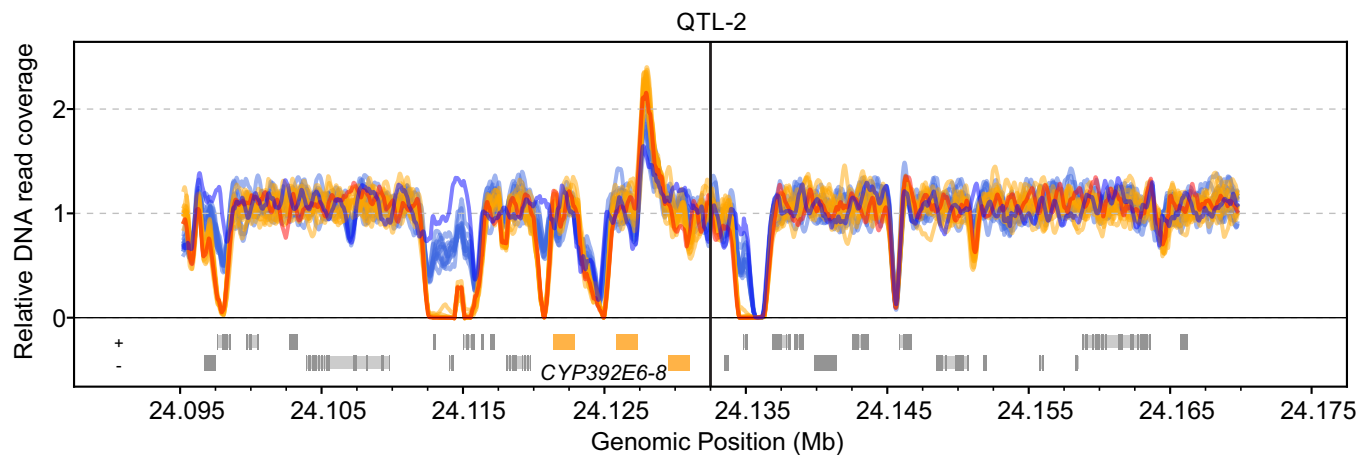

Supplement: S4 Fig — Vertical lines indicate the locations of averaged BSA peaks. Candidate genes CYP392A16 (A) and CYP392E6-8 (B) are highlighted in yellow. Otherwise, coding exons and introns are depicted as dark gray and lighter gray boxes, respectively. Symbols + and–denote forward and reverse gene orientations. Coverage is color-coded according to treatment and strain (legend, top). (PDF) [file pgen.1009422.s004.pdf]

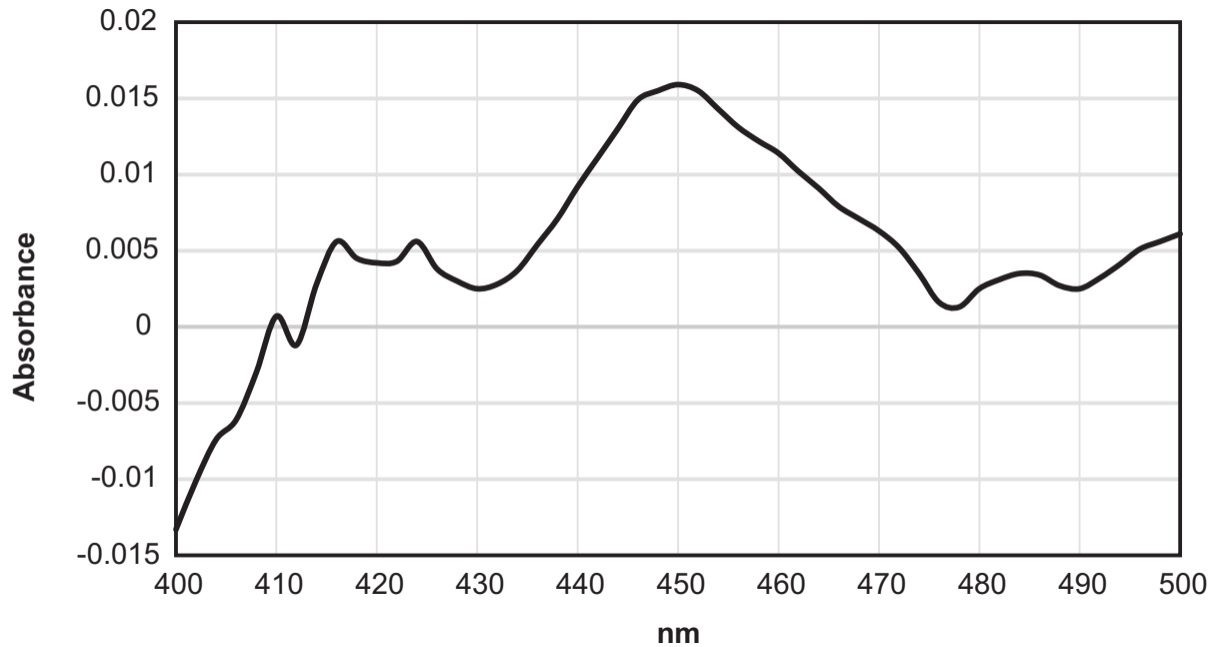

Supplement: S5 Fig — (PDF) [file pgen.1009422.s005.pdf]
