# Supplementary material for: High-resolution genetic mapping reveals cis-regulatory and copy number variation in loci associated with cytochrome P450-mediated detoxification in a generalist arthropod pest
Source: PLoS Genet. 2021 Jun 21;17(6):e1009422. doi: 10.1371/journal.pgen.1009422 (PMC8248744; doi:10.1371/journal.pgen.1009422)
Supplement: S1 Table — (DOCX) [file pgen.1009422.s006.docx]

**S1 Table.** The toxicity of pyflubumide in different susceptible and resistant strains and their crosses

| **Strain** | **F1 LC_50_ (95% CI) (mg/L)** | **Slope ± SE** | **RR (95% CI)** | **D** |
| --- | --- | --- | --- | --- |
| Wasatch ♀ | 3.08 (2.76 - 3.36) | 8.69 ± 0.86 | 1 | - |
| JP-S ♀ | 5.08 (4.88 - 5.28) | 7.64 ± 0.53 | 1.65 (1.55 - 1.75) | - |
| JPR-R1♀ | 1373.72 (1168.46 - 1585.97) | 1.76 ± 0.15 | 445.64 (379.81 - 522.89) | - |
| JPR-R2 ♀ | 1978.87 (1739.93 - 2198.21) | 3.56 ± 0.34 | 641.95 (566.60 - 727.33) | - |
| JPR-R1♀ × JP-S ♂ | 21.64 (19.03 - 23.96) | 4.02 ± 0.37 | 7.02 (6.21 - 7.94) | -0.51 |
| JP-S ♀ × JPR-R1 ♂ | 18.16 (16.28 - 19.93) | 3.66 ± 0.30 | 5.89 (5.27 - 6.58) | -0.57 |
| JPR-R2 ♀ × Wasatch ♂ | 7.68 (7.04 - 8.34) | 4.09 ± 0.37 | 2.49 (2.26 - 2.74) | -0.72 |
| Wasatch ♀ × JPR-R2 ♂ | 6.54 (5.96 - 7.11) | 4.83 ± 0.40 | 2.12 (1.95 - 2.31) | -0.77 |
| Concentration-mortality data of pyflubumide was obtained from adult females. CI, confidence interval; D, degree of dominance; RR, resistance ratio. | | | | |
