# Supplementary material for: High-resolution genetic mapping reveals cis-regulatory and copy number variation in loci associated with cytochrome P450-mediated detoxification in a generalist arthropod pest
Source: PLoS Genet. 2021 Jun 21;17(6):e1009422. doi: 10.1371/journal.pgen.1009422 (PMC8248744; doi:10.1371/journal.pgen.1009422)
Supplement: Attachment — Submitted filename: Response_to_Reviewers_final.docx [file pgen.1009422.s009.docx]

Kirsten Bomblies
Section Editor: Evolution
PLOS Genetics

The comments and suggestions from two expert reviewers are now available. Both reviewers agree that the study is exciting and contributes significantly to our understanding of the genetic basis of resistance in a major pest. The results are compelling and the writing is clear and concise. The reviewers have minor suggestions to improve clarity and expand the discussion someone. One additional thing the authors may wish to consider- if this is the first case of a CNV underlying resistance, the authors could amplify the novelty of this message somewhat.

**RESPONSE.** Dear editors and reviewers, we thank you for your support and constructive comments.

A few previous studies have collected evidence that link CNV of detoxification genes and target-sites with pesticide resistance, and we have referenced these publications in the introduction (e.g. line 70-73 and 85-87) and discussion (e.g. line 359-360). However, to the best of our knowledge, this is the first report of a resistance phenotype that is underpinned by CNV of a *CPR* gene, a vital component of cytochrome P450 metabolism, and this could be considered a new type of resistance mechanism. After discussing among the co-authors, we feel that we have placed sufficient emphasis on this newly discovered molecular mechanism throughout the manuscript (including as a focal point in both the title and abstract).

We have listed the additional responses point-by-point in purple below and feel that the manuscript has been greatly improved by addressing the reviewer’s comments. We hope that our revised manuscript is now acceptable for publication in PLOS Genetics.

Reviewer's Responses to Questions

**Comments to the Authors:
Please note here if the review is uploaded as an attachment.**

Reviewer #1: The aim of the study is to identify the causes of resistance to a new acaricide, pyflubumide, in populations of Tetranychus urticae. The authors show that resistance is multifactorial, they identify three main QTLs that contain detoxification genes. Among these detoxification genes, a P450, CYP392A16, is capable of metabolizing the active deacylated form of the insecticide into a less toxic metabolite. This work represents a large body of results and is essential in understanding resistance mechanisms and managing a major pest. However, I believe that some improvements to the manuscript are possible, please see details below.

Major points
Results
- Paragraph “Candidate genes for pyflubumide resistance are linked to cytochrome P450-mediated detoxification”, from line 196 to 208, I am surprised that the authors didn’t do any comments on the gene “aryl hydrocarbon receptor interacting protein” which is down-regulated in QTL1. In mammals, this gene codes for a chaperone protein which binds to inactive Aryl Hydrocarbon Receptor (AhR) in the cell cytoplasm. AhR is known in arthropod to regulate the expression of detoxification genes. As a general comment for this manuscript, I would say that although I can understand that the authors have other works going on to characterize the cis-regulation involved in the up-regulation of P450s in their resistant populations, I found that discussion regarding this specific point is missing. Comparison between the promoter of CYP392A16 between susceptible and resistant population/mites is information available in author hands.

**RESPONSE.** To clarify, the *T. urticae* gene described as “aryl hydrocarbon receptor interacting protein” in Supplementary Data S2 was not significantly down-regulated in the pyflubumide-selected populations according to our significance thresholds. The log_2_FC was -0.117 and the adjusted *p*-value was 0.120. Additionally, the functional description of the gene has been based on automated reciprocal BLAST-searches and a thorough focused investigation is lacking. Although searches in the Pfam-A database only gave a significant hit to tetratricopeptide repeat (TPR_1 (PF00515), 5.3e^-07^), the association between the *T. urticae* protein and aryl hydrocarbon receptor was supported by the Interpro/PTHR databases. Here, the protein was classified as a member of the AIP/AIPL1 (IPR039663) family that includes aryl hydrocarbon receptor-interacting proteins. These are known to interact with Hsp90, a chaperone of aryl hydrocarbon receptors in mammals.

Even though no significant difference in transcription, nor any non-synonymous SNPs were observed for this gene in our focal populations, we agree with the reviewer that a tentative description of this candidate regulator in QTL-1 would benefit the manuscript. Lines 216 – 223 now read:

The ~75 kb genomic interval of QTL-1 also included *tetur06g04470*, a gene that codes for a protein that is classified as a member of the AIP/AIPL1 family (IPR039663), a group of proteins that include aryl hydrocarbon receptor (AhR)-interacting proteins. AhR participates in a signaling pathway that induces the transcription of detoxification genes in insects and changes in this pathway can promote pesticide resistance [43,44]. However, no significant differential transcription, nor non-synonymous single-nucleotide differences were observed for *tetur06g04470,* suggesting that *tetur06g04470* likely does not contribute to pyflubumide resistance in our strains.

Promotor analysis, or the identification of nearby transcriptional enhancers, is not trivial in non-model organisms that lack the tools for experimental validation. The few cases where convincing promotor related *cis*-effects were documented in insects either consisted of drastic structural changes, such as the insertion of an *Accord* transposable element into the five prime end of the *Cyp6g1* gene of *Drosophila melanogaster* (Daborn *et al.*, Science 2002) or required extensive functional validation such as for the genetic polymorphisms upstream of the *CYP6P9a* gene of *Anopheles funestus* (Weedall *et al*., Science Transl. Med. 2019). Our current study, and our previous work with *T. urticae*, have revealed that there is substantial variation between spider mite strains (single-nucleotide changes every ~90 bp, and indels in introns and between genes are very common), and it is unknown in mites over which distances transcriptional enhancers can function (that is, how far from promoters to look). It is true that such polymorphisms could potentially be linked to changes in putative transcription factor binding sites, if they were available. However, the identification of candidate transcription factor binding sites using a computational approach would rely on insect motif databases and likely create a substantial bias in our findings (this is currently a black box in spider mites). As such, our findings would be highly speculative in the absence of extensive experimental validation, for example by reporter gene assays, an approach that has been successfully used for a few insects. Given the lack of available mite-derived cell lines, such approaches are currently not trivial for *T. urticae.* Although some other methods to validate the interaction of candidate regulatory sequences could be tried, they would require substantial development. While we are very interested in enabling such future studies in spider mites, we feel they are beyond the scope of what we can do for the current study.

However, we certainly appreciate the reviewer’s comment, and we have incorporated some of these considerations in the Discussion. Lines 343 – 349 now read:

In insects, transcription of detoxification genes is controlled by a number of regulatory pathways, including the AhR/ARNT and CncC/Keap1 cascades, in which *cis*-acting transcription factor binding sites have been identified [2,43]. In contrast, transcriptional regulation remains poorly understood in mites, and promotor/enhancer analyses are currently not trivial to conduct or interpret. Future studies are therefore needed to test our hypothesis of *cis*-regulation, and to identify the potential *cis*­-acting elements and their transcription factor binding partners, using suitable experimental set-ups [60,61].

Discussion
- From line 324 to 329, in the same idea than the previous comment, the discussion regarding cis-regulation is very limited. Ok for “Future studies are needed to test the hypothesis of cis-regulation using a more suitable experimental set-up”, however the authors can at least say a few words on the already known mechanisms in arthropods (transcription factors involved).

**RESPONSE.** We have addressed this issue alongside the previous comment raised by the reviewer. Please see above.

Materials & methods
- Lines 397-398, “JPR-R1 and JPR-R2 were selected for high levels of pyflubumide resistance from the JP-R strain”, I understand that the authors don’t want to repeat what was already published, but I have to read the reference 41 to understand the difference between JPR-R1 and JPR-2. As most of the results of the manuscript are on these two strains, maybe the authors can add one more sentence to explain at least the selection way.

**RESPONSE.** We have expanded the description of our two resistant strains. The lines now read:

The JPR-R1 and JPR-R2 strains were selected for high levels of pyflubumide resistance from the JP-R strain with two different laboratory selection regimes. JPR-R1 was first selected by spraying pyflubumide on detached mite-infested bean leaves for two generations. Subsequently, both JPR-R1 and JPR-R2 were selected by feeding on pyflubumide sprayed potted bean plants (100 mg/L of pyflubumide) [41].

Figure 5: I find it frustrating not to have the names of the genes identified in the QTL regions (despite the fact that they are mentioned in the supplementary data S2). The authors could give names of P450 and CPR in smaller sizes and indicate the names of the other genes shown in the figure.

**RESPONSE.** We understand this comment, and we tried to incorporate the reviewer’s recommendations. Unfortunately, we found that we could not add the names of the neighbouring genes within the 75 kb genomic intervals due to the font size limitations imposed by PLOS Genetics (Arial, Times, or Symbol font only in 8-12 point).

Supplementary Data S2
- In the genome-wide transcription, there are many detoxification genes given without a precise name. As far as I know all detoxification genes were annotated in the genome of Tetranychus urticae, so why is there still gene defined as: “Cytochrome P450”, “Carboxyl/cholinesterase”, etc….

**RESPONSE.** Thank you for bringing this to our attention. Missing identifiers of the manually annotated detoxification genes of *T. urticae* have been added to Supplementary Data S2. To clarify, several fragmented or pseudogenized genes have been found for most of the canonical detoxification multi-gene families in *T. urticae* and some gene copies have not been given specific identifiers in previous work.

Minor points:
- Line 293, please correct pyflubmide by pyflubumide.
**RESPONSE.** Line 293 is corrected.
- Line 631, correct meabolite by metabolite.
**RESPONSE.** Line 631 is corrected.
- Ref 9, add the five following names and et al. after Daborn PJ because he was not the sole author of this publication.
**RESPONSE.** Reference 9 is corrected.
- Ref 19, the title appears two times, correct it.
**RESPONSE.** Reference 19 is corrected.
- Ref 41, remove [cited 9 Mar 2020] and add volume and page number.
**RESPONSE.** Reference 41 is corrected.
- Please recheck all the references (species names in italic, duplicated title, sometimes the name of the journal is in full sometimes in abbreviated form…).
**RESPONSE.** We have gone through the reference list and corrected these errors. We thank the reviewer for the detailed comments on the references that caught a number of needed corrections.

Reviewer #2: This is a very nice study identifying the genetic determinants of pyflubumide resistance in spider mites, using genetic crosses, geomics, transcriptomics and functional expression. The methods are explained in detail and the results are convincing. I have only minor comments.

lines 177 and 484: In both the results and the methods, the method for obtaining a p-value for a given window is described only with a citation (and a mention, in the results, that it involves permutations). Please include a very brief description (one or two sentences) of the method.

**RESPONSE.** We have included a brief description of the permutation-based approach in the methods section. Lines 514-522 read:

Significant responses to pyflubumide selection were detected using a permutation-based approach as described in Wybouw *et al*. [29]. The genome-wide allele frequency differences were permuted for the 11 pairs of selected/control populations 10^4^ times and were averaged across all pairs per permutation. For these analyses, the three chromosomes were concatenated and made circular to maintain linkage information (the end of the third chromosome was connected with the beginning of the first chromosome). Using the 10^4^ permutations, the distribution of the absolute values of the maximal deviations in the averaged allele frequency differences was calculated and QTLs were assigned using an FDR of 0.05.

lines 183-185: I found this sentence confusing, and it left me wondering how many "a number" was. I suggest rephrasing as "where the signal came close to significance", or replace "a number" with something a little more descriptive (eg: "most", or give respective numbers of replicates in which elevations were found).

**RESPONSE.** We have followed the advice of the reviewer and adjusted the sentence. Lines 184-186 now read:

We also noted two additional genomic regions where the allele frequency differences nearly exceeded the significance threshold (hereafter referred to as subsidiary peaks 1 and 2) (Fig 4C).

lines 189-190: I don't understand the description of QTL genomic intervals at all. Please rephrase this more clearly. Please also state the size of each QTL genomic interval, as defined here.

**RESPONSE.** We have rephrased the description of these intervals and have specified the interval sizes. The manuscript now reads:

Of the 413 differentially expressed genes (DEGs), 123 (30.8%) were within the three top QTL genomic intervals defined as the regions with allele frequencies similar to those of the peaks (at least one of five consecutive windows having allele frequency values within 0.005 of the peak values). Specifically, 24 DEGs were located within the top 180 kb QTL-1 region, whereas 25 and 74 DEGs were within the 130 kb QTL-2 and 110 kb QTL-3 genomic intervals, respectively.

lines 188-193: I do not understand how to interpret this result.. With the exception of large CNVs, I don't see why differentially-expressed genes should cluster within QTLs. Each QTL is presumably driven by one (or a few) mutations affecting the expression or sequence of just a handful of genes. Here, the authors argue that 123 genes DEGs are all clustered within these QTLs. At the end of the paragraph, the authors then argue that the 290 DEGs that do not fall within the 3 QTLs are trans-regulated from within the three QTLs, which would require yet more processes under selection, all falling within the same three regions. This all seems rather unlikely to me and, if true, it's not clear to me how this would occur. Would it be possible to include a statistical analysis of whether DEGs are significantly more likely to be found within a QTL than outside of one (the analysis would need to take into account the overall gene-richness of the QTL regions compared to the genome as a whole)? We are told that 30% of the DEGs are found within the QTLs, but what proportion of all genes are in the QTLs?

**RESPONSE.** We believe there has been a significant misunderstanding here as a result of our admittedly unclear explanation of the expectation and the observed patterns.

First, yes, factually there is a clear “overrepresentation” of DEGs in the QTL intervals (the QTL regions only represent ~0.5% of the entire *T. urticae* genome but harbor ~30% of the DEGs; *p*-value < 2.2e-16, chi-square test, R version 3.4.3, assuming an equal gene density across the *T. urticae* genome, a fair assumption given the most recent genome annotation and structure, Wybouw & Kosterlitz *et al.,* 2019 Genetics).

However, the critical point is that the finding results from the specific nature of our experimental design, which is a differential gene expression analysis between selected and unselected populations. Briefly, in performing a differential gene expression analysis between our selected and unselected populations, it is ONLY in the genomic regions of genetic differentiation – the three well-supported QTL regions – that contrasting haplotypes can be assessed, and any *cis*-differences can be inferred. In the non-QTL regions, the frequencies of haplotypes from the two BSA parents are not significantly different between selected and unselected populations, and therefore no *cis*-effects are expected to be detectable in our experimental set up. So, again, the detection of many DEGs within the QTL intervals is actually exactly what was expected, but it reflects the experimental set-up and not an unexpected biological phenomenon.

Alternatively, the genes with different RNA levels that are not linked to the QTL regions are presumably *trans*-regulated by variants that genetically distinguish the selected and unselected populations (that is, the three QTL intervals).

However, to be very clear we do not expect that all (or even all but a few) DEGs within the QTL regions are associated with pyflubumide resistance. Within the QTL intervals, the large number of DEGs is just an impact of linkage in our experimental set-up (the close proximity to the causal allele(s) in one or a small number of genes causal for resistance). Likewise, there is no expectation that *trans* “DEGs” are related to detoxification. We were careful to not make such claims in the manuscript, as they would obviously not be true (but there does need to be a sentence or two about the findings, which did turn out to be important, see below).

What the differential gene expression analysis did accomplish for our study is that it identified candidate genes within the QTL intervals that have different expression levels depending on the contrasting haplotypes contributed by each BSA parent. We reasoned that one (or a few) of these DEGs might be causal for resistance, and the transcriptional change might implicate a molecular mechanism (*cis* variation in expression). This is what helped lead us to, for example, both *CYP392A16* and *CPR* as candidates to underlie resistance (please see Section “Candidate genes for pyflubumide resistance are linked to cytochrome P450-mediated detoxification”). And, the DEG analysis, along with additional data, did help to suggest a molecular mechanism in the case of these genes.

Given our wording, we understand why this section was confusing; we have now tried to make the expectations and our tentative observations for the DEG analysis more clear in the revised manuscript. Lines 194-201 now read:

The linkage information suggests that *cis*-regulation could play a dominant role in the different expression levels of these 123 DEGs (note that in the three QTL regions there are large differences in the frequency of the haplotypes from the two parents between the selected and unselected replicates, so *cis*-expression QTLs (eQTLs) are expected to be uncovered compared to distant genomic locations where haplotype frequencies are not significantly different). The RNA levels of the other (QTL-interval distant) 290 DEGs are presumably *trans*-regulated, with the different RNA levels controlled by genetic variants within the three QTL regions that genetically differentiate the selected and control populations.

lines 202 and 211: the numbers of DEGs described for the intervals here are different to the numbers listed on lines 190-191, presumably because the "interval" used is different. I found this confusing again, perhaps because I could not undertand lines 189-190.

**RESPONSE.** The numbers of DEGs were indeed different because the genomic intervals were defined differently. As outlined in the original manuscript at lines 188-189 (revised manuscript at lines 190-191), the first set of DEGs was identified in the relatively broad top QTL intervals. As outlined in the original manuscript at lines 197-198 (revised manuscript at lines 206-207), we subsequently focused on the ~75 kb genomic intervals that bracket the average of the BSA peaks. This focus was motivated by previous work that uses similar BSA experimental designs and revealed that the causal variants lied within tens of kb from the BSA peak.

line 415: It seems to me that this is only the expected dose-response for monogenic resistance when resistance is completely recessive. I can't see a strong justification for using this as a fair test of the null hypothesis of monogenicity.

**RESPONSE.** This test is often employed to test for a monogenic recessive genetic basis of pesticide resistance (see for instance Van Leeuwen *et al*., 2012, PNAS and Van Leeuwen *et al*., 2004, EAA). The test is not dependent on the mode of inheritance, as it uses the observed dose-response data of the F1 generation (whether dominant or recessive). Of course, the F1 response determines which parent is used for back-crossing. We have re-formulated the sentence which now reads:

“The expected dose-response relationships for F2 females for monogenic recessive resistance was calculated using c = 0.5 W (F1) + 0.5 W (JPR-R strain), where c is the expected response (mortality) at a given concentration and W is the observed response (mortality) of the parents at the respective concentration”

Figure 1: I don't understand what the "monogenic" lines are showing? As they the theoretical predictions from the equation on line 415?

**RESPONSE.** Yes, these are the predicted values if a monogenic recessive genetic basis is assumed. We had specified this distinction in the figure legend. To avoid any potential confusion, we have now also added “(black versus white triangles, respectively)” to ensure the reader can more easily distinguish between the observed and predicted values.
